# Inherited *BRCA1* and *RNF43* pathogenic variants in a familial colorectal cancer type X family

**DOI:** 10.1007/s10689-023-00351-2

**Published:** 2023-12-08

**Authors:** James M. Chan, Mark Clendenning, Sharelle Joseland, Peter Georgeson, Khalid Mahmood, Jihoon E. Joo, Romy Walker, Julia Como, Susan Preston, Shuyi Marci Chai, Yen Lin Chu, Aaron L. Meyers, Bernard J. Pope, David Duggan, J. Lynn Fink, Finlay A. Macrae, Christophe Rosty, Ingrid M. Winship, Mark A. Jenkins, Daniel D. Buchanan

**Affiliations:** 1 Colorectal Oncogenomics Group, Department of Clinical Pathology, Melbourne Medical School, Victorian Comprehensive Cancer Centre, The University of Melbourne, 305 Grattan Street, Parkville, VIC 3010 Australia; 2 Centre for Cancer Research, University of Melbourne, The University of Melbourne, Parkville, VIC Australia; 3 Melbourne Bioinformatics, The University of Melbourne, Melbourne, VIC Australia; 4 Quantitative Medicine and Systems Biology Division, Translational Genomics Research Institute (TGen), Phoenix, AZ USA; 5 Faculty of Medicine, Frazer Institute, The University of Queensland, Brisbane, QLD Australia; 6 Australian Translational Genomics Centre, Queensland University of Technology, Brisbane, QLD Australia; 7 Colorectal Medicine and Genetics, Royal Melbourne Hospital, Parkville, VIC Australia; 8 Genomic Medicine and Family Cancer Clinic, Royal Melbourne Hospital, Parkville, VIC Australia; 9 Envoi Pathology, Brisbane, QLD Australia; 10 School of Medicine, University of Queensland, Herston, QLD Australia; 11 Department of Medicine, The University of Melbourne, Parkville, VIC Australia; 12 Centre for Epidemiology and Biostatistics, The University of Melbourne, Melbourne, VIC Australia

**Keywords:** Colorectal cancer, Serrated polyposis syndrome, FCCTX, Digenic inheritance, *BRCA1*, *RNF43*, Germline pathogenic variant

## Abstract

Genetic susceptibility to familial colorectal cancer (CRC), including for individuals classified as Familial Colorectal Cancer Type X (FCCTX), remains poorly understood. We describe a multi-generation CRC-affected family segregating pathogenic variants in both *BRCA1*, a gene associated with breast and ovarian cancer and *RNF43*, a gene associated with Serrated Polyposis Syndrome (SPS). A single family out of 105 families meeting the criteria for FCCTX (Amsterdam I family history criteria with mismatch repair (MMR)-proficient CRCs) recruited to the Australasian Colorectal Cancer Family Registry (ACCFR; 1998–2008) that underwent whole exome sequencing (WES), was selected for further testing. CRC and polyp tissue from four carriers were molecularly characterized including a single CRC that underwent WES to determine tumor mutational signatures and loss of heterozygosity (LOH) events. Ten carriers of a germline pathogenic variant *BRCA1*:c.2681_2682delAA p.Lys894ThrfsTer8 and eight carriers of a germline pathogenic variant *RNF43*:c.988 C > T p.Arg330Ter were identified in this family. Seven members carried both variants, four of which developed CRC. A single carrier of the *RNF43* variant met the 2019 World Health Organization (WHO^2019^) criteria for SPS, developing a *BRAF* p.V600 wildtype CRC. Loss of the wildtype allele for both *BRCA1* and *RNF43* variants was observed in three CRC tumors while a LOH event across chromosome 17q encompassing both genes was observed in a CRC. Tumor mutational signature analysis identified the homologous recombination deficiency (HRD)-associated COSMIC signatures SBS3 and ID6 in a CRC for a carrier of both variants. Our findings show digenic inheritance of pathogenic variants in *BRCA1* and *RNF43* segregating with CRC in a FCCTX family. LOH and evidence of BRCA1-associated HRD supports the importance of both these tumor suppressor genes in CRC tumorigenesis.

## Introduction

Colorectal cancer (CRC) has one of the highest rates of aggregation within families (familial CRC), with up to 35% of CRC thought to be caused by inherited genetic risk factors [1]. However, the underlying cause of CRC can be assigned to one of the inherited CRC and polyposis syndromes in only 5–10% of cases [2], therefore, the genetic cause of the majority of familial CRC remains unknown. The term Familial Colorectal Cancer Type X (FCCTX) was proposed to define families with a strong CRC family history that meet the Amsterdam I criteria [3] where the tumors are DNA mismatch repair (MMR)-proficient/microsatellite stable and do not carry a germline pathogenic variant in one of the MMR genes (Lynch syndrome) [4, 5]. The genetic factors underlying FCCTX are poorly understood and are likely to be heterogeneous involving multiple susceptibility genes [6].

Serrated polyposis syndrome (SPS) is characterized by the presence of multiple serrated colorectal polyps (hyperplastic polyp, sessile serrated lesion (SSL) and traditional serrated adenoma) resulting in an increased risk of developing CRC [7–9]. The diagnostic criteria for SPS was re-defined by the World Health Organization (WHO) in 2019 [10] to include (i) 5 or more serrated polyps proximal to the rectum, all 5 mm or greater in size, with 2 or more 10 mm or greater in size or (ii) more than 20 serrated polyps of any size in the large bowel, with 5 or more proximal to the rectum. The progression from serrated polyp to carcinoma, referred to as the serrated neoplasia pathway of tumorigenesis, is characterized by distinct molecular features, including the presence of microsatellite instability (MSI), high levels of the CpG island methylator phenotype (CIMP) and somatic mutations in the oncogenes *BRAF* or *KRAS* [11]. However, the genetic etiology of SPS remains poorly understood [12, 13]. Recently, germline pathogenic variants in *RNF43* have been proposed to underlie SPS [14–18], but they account for only a small proportion of cases [19]. As such, expert groups are yet to recommend the inclusion of *RNF43* in multi-gene testing panels for patients with SPS [12].

The *BRCA1* gene acts as a tumor suppressor through its role in DNA repair [20]. Germline pathogenic variants in *BRCA1* confer high risks of breast and ovarian cancers [21]. The association between *BRCA1* pathogenic variants and CRC development is more uncertain [22]. Multiple studies have investigated whether carriers of germline *BRCA1* pathogenic variants have an increased risk of developing CRC, with mixed results [23, 24].

It has been suggested that digenic inheritance may account for some cases of familial CRC and polyposis syndromes, however there are few reports in the literature [25–27]. In this study, we describe a family meeting FCCTX criteria where multiple cancer-affected individuals carried germline pathogenic variants in both the *BRCA1* and *RNF43* genes on chromosome 17q. The tumor characteristics from carriers were assessed to characterize the drivers of tumorigenesis. Our findings demonstrate a possible role for digenic inheritance in the predisposition to familial CRC.

## Methods

### Study cohort

The family presented was identified from the Australasian Colorectal Cancer Family Registry (ACCFR) (HREC:13,094) [28–30]. The ACCFR recruited multiple-member CRC-affected families from Family Cancer Clinics across Australia and New Zealand between 1998 and 2008. Participants provided written consent to access their tumor tissue and provided a blood sample [30]. Methodology for germline MMR and *MUTYH* gene testing and tumor characterization have been described previously [28].

### Germline sequencing and variant detection

CRC-affected individuals 009 and 014 had germline whole exome sequencing (WES) performed. Briefly, 50ng of genomic DNA was fragmented to an average size of 180 bp in length using a Covaris focused-ultrasonicator (Covaris, Woburn, MA, USA). An Illumina sequencing technology compatible whole genome library was created using Kapa Biosystems Hyper Prep Kits (Kapa Biosystems Inc., Wilmington, MA, USA). These libraries were then subjected to whole exome target enrichment using Agilent SureSelect hybrid capture version 4 kits (Agilent Technologies, Santa Clara, CA, USA). Parallel sequencing of libraries was performed on Illumina HiSeq2000/2500 system using version 1.5 or version 3 chemistry using paired-end 2 × 100 bp reads (Illumina, San Diego, CA, USA). All sequencing reads were converted to industry standard FASTQ files using BCL2FASTQ v1.8.4. FASTQ files were processed using a pipeline based on industry standard software packages and programs. Sequencing reads were aligned to the GRCh37 human genome reference using v0.7.8 BWA-MEM aligner [31] to generate BAM files. SAMtools v0.1.19 [32] was used to sort BAM files and Picard v1.111 (http://broadinstitute.github.io/picard/) to mark duplicate read pairs. Post alignment joint insertion/deletion (indel) realignment and base quality scores recalibration was performed on the BAM files using GATK v3.1-1 [33]. Variants were called from germline BAM files individually using GATK Haplotype Caller v3.1-1 [34] and SAMtools v0.1.19 [35].

### Germline variant annotation

Germline variants were annotated with the Ensembl Variant Effect Predictor release 105 (December 2021) for the human genome reference GRCh37 including the CADD predicted pathogenicity scores for each variant [36, 37]. The RefSeq transcript NM_007300.4 was used for *BRCA1*. The RefSeq transcript NM_017763.5 was used for *RNF43*. Sanger sequencing of the *BRCA1* and *RNF43* pathogenic variants was used for confirmation of the variants in persons 009 and 014 and to segregate the variants in 19 other family members with available DNA.

### Tumor tissue sample processing and nucleic acid preparation

Where available, formalin-fixed paraffin-embedded (FFPE) tumor tissue blocks were obtained. MMR status was determined with immunohistochemistry as previously described [28]. Sections were stained with haematoxylin and eosin and prepared for pathological review. Tumor, polyp and histologically normal mucosa were macrodissected and processed independently. DNA was extracted with the QIAamp DNA FFPE Tissue kit following standard protocols (Qiagen, Hilden, Germany).

### Tumor tissue sequencing and variant detection

CRC tumor tissue and matched blood-derived DNA from person 009 were prepared according to the procedure for Hybridization Capture using the Agilent SureSelectXT Low Input Clinical Research Exome v2 kit. The prepared libraries were sequenced with Illumina sequencing technology comprising 150 bp paired. Raw FASTQ files underwent adapter sequence trimming using trimmomatic v.0.38 [38] and alignment to the human genome reference GRCh37 using BWA v.0.7.12 [31]. Duplicate reads were identified with Picard v2.8.2. Mean on target coverage for the tumor and buffy coat samples was 499.3 and 79.5 respectively. Germline variants were called with HaplotypeCaller from GATK 4.0.0 [39] using GATK’s recommended workflow. Somatic single-nucleotide variants (SNVs) and short insertions and deletions (indels) were called with Mutect2 [40] with the recommended GATK practices and Strelka v.2.9.2 [41] with Illumina’s recommended workflow. Mutations reported by both callers were filtered to PASS variants with a minimum variant allele frequency of 0.1 and minimum depth of 50 reads.

### Tumor loss of heterozygosity analysis

Two CRC and two polyp tissue DNA samples from two carriers of both variants were assessed for loss of heterozygosity (LOH) of the wildtype alleles of the *BRCA1* and *RNF43* variants using standard Sanger sequencing protocols. Short (179 bp) *BRCA1* amplicons were generated using GCAGAAGAGGAATGTGCAACATTCT and TTATCTTTCTGACCAACCACAGGAA with sequencing occurring in the reverse direction. Short (182 bp) *RNF43* amplicons were generated using ACAGGCTACTCAGGGTCAAATAGAT and CGAATGAGGTGGAGTCTTCGA with sequencing occurring in the forward direction. Tumor tissue DNA was available for a single CRC from person 009 for extended LOH assessment using WES tumor data. The captured regions of the genome were assessed for evidence of LOH by interrogating heterozygous germline variants in the tumor for their presence as homozygous reference or homozygous alternative in the tumor tissue. A tumor cellularity estimate of 80% was used. Germline variants with an allele frequency of between 0.4 and 0.6 were considered heterozygous. An allele frequency difference of 0.3 or greater in the somatic tissue, limited to variants with a germline depth ≥ 10 and tumor depth ≥ 30, was considered evidence of LOH. Individual variants suggesting the presence of LOH were aggregated to determine likely genomic regions of LOH. The algorithm used is available at https://github.com/supernifty/LOHdeTerminator.

### Tumor mutational signature analysis

SNVs and indels were filtered to those in the capture region. These filtered SNVs and indels were used to calculate tumor mutational signatures according to the method given by SignatureEstimation [42] from the set of COSMIC version 3.2 signatures [43] limited to signatures observed in CRC tissue comprising 15 single base substitution (SBS) signatures and 5 indel (ID) signatures [44] as commonly recommended [45], including SBS3 and ID6 given their association with *BRCA1* mutations. SBS3 or ID6 present at > 10% or > 20% proportion in the tumor signature profile, respectively, was considered positive for defective homologous recombination-based DNA damage repair (HRD).

## Results

**Fig. 1:**
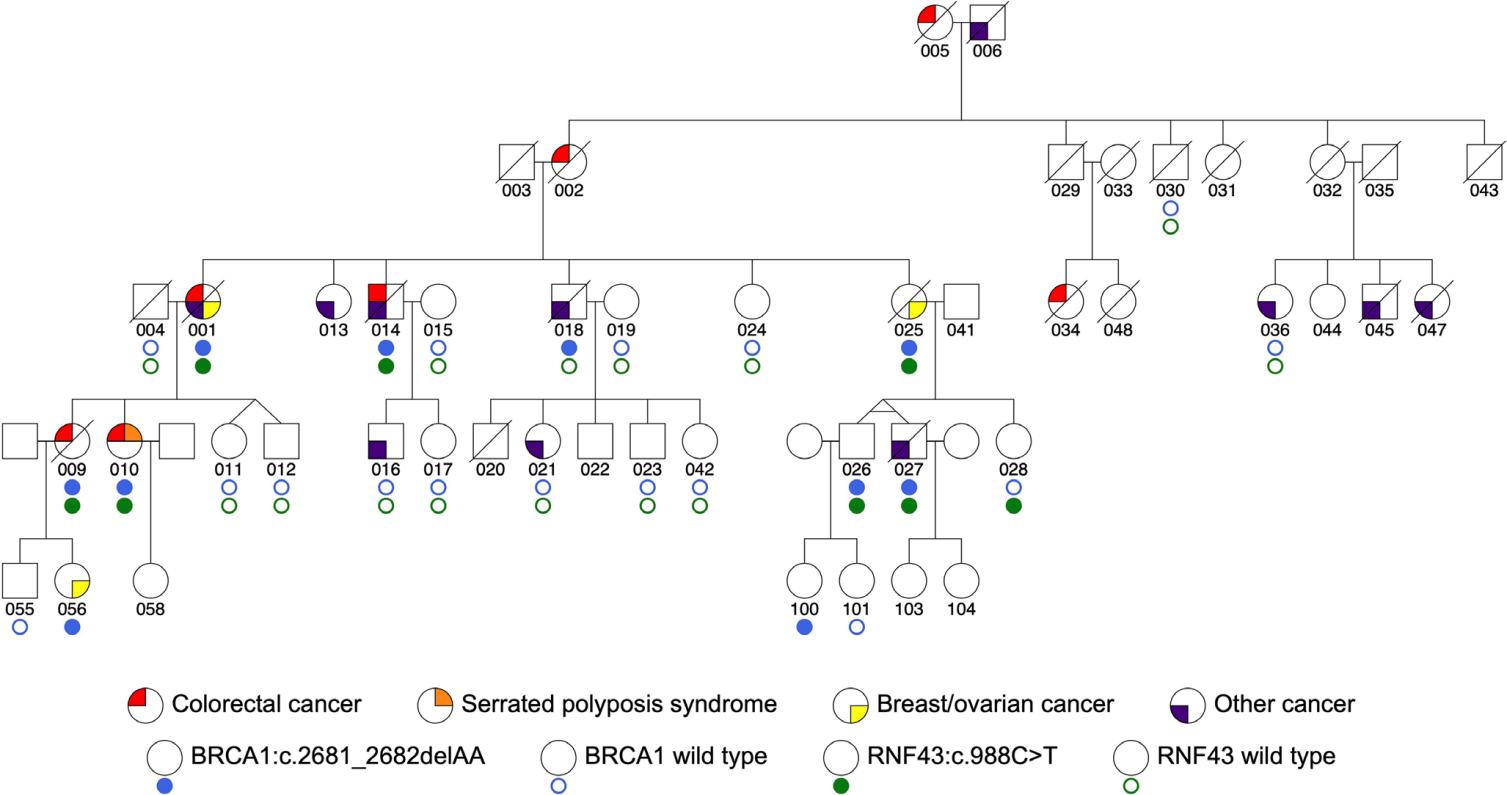
Pedigree diagram for a family with colorectal cancer, serrated polyposis syndrome and *BRCA1*:c.2681_2682delAA and *RNF43*:c.988 C>T germline pathogenic variants. The indicated carriers include obligate carriers

Two germline pathogenic variants were identified; one in *BRCA1*:c.2681_2682delAA, a frameshift pathogenic variant located in exon 10 encoding p.Lys894ThrfsTer8, and another in *RNF43*:c.988 C>T, a nonsense pathogenic variant located in exon 9 encoding p.Arg330Ter, in one family meeting the FCCTX criteria. The family pedigree with cancer-affected and carrier status is shown in Fig. 1. No other loss of function or predicted pathogenic variants were identified in established hereditary CRC and polyposis genes. Ten individuals carried the *BRCA1*:c.2681_2682delAA variant and eight individuals carried the *RNF43*:c.988 C>T variant. Seven individuals carried both pathogenic variants of whom six were cancer-affected (4 CRC, 1 breast/ovarian cancer, 1 metastatic cancer of unknown primary). All four of the CRC-affected relatives tested carried both pathogenic variants. Only a single carrier of both variants was cancer-unaffected at age 58 (person 026). Where both variants were tested, two individuals were found to carry only a single variant, person 018 carried only the *BRCA1* variant and person 028 carried only the *RNF43* variant, where each likely represents a separate homologous recombination event on chromosome arm 17q. Details of carrier status and their tumors are provided in Table 1.

**Table 1:** Cancer and colonic polyp history together with the carrier status of the *BRCA1*:c.2681_2682delAA and *RNF43*:c.988 C>T germline pathogenic variants in people from a family meeting FCCTX criteria

Person	Sex	*BRCA1*:c.2681_2682delAA	*RNF43*:c.988 C>T	Age at diagnosis	Tumor type	Tumor location	Tumor histologic type
001	F	Carrier	Carrier	53	CRC	Caecum	Adenocarcinoma
				62	Peritoneal	NA	NA
				63	Ovarian	NA	NA
002	F	unknown	unknown	58	CRC	Colon	Adenocarcinoma
005	F	unknown	unknown	67	Intestinal	NA	NA
006	M	unknown	unknown	80	Laryngeal	Larynx	NA
009	F	Carrier	Carrier	44	CRC	Transverse colon	Adenocarcinoma
010^a^	F	Carrier	Carrier	44	Colonic polyp	Ascending colon	Tubular adenoma
				56	CRC	Sigmoid colon	Adenocarcinoma (15 mm) (background of SSL on histology)
				56	Colonic polyp	Transverse colon	Hyperplastic polyp (10 mm)
				59	Colonic polyp	Ascending colon	Adenomatous polyp (6–8 mm)
				59	Colonic polyp	Ascending colon	SSL (6–8 mm)
				59	Colonic polyp	Sigmoid colon	2 hyperplastic polyps (6–8 mm)
				62	Colonic polyp	Rectum	Hyperplastic polyp (5–8 mm)
011	F	Wildtype	Wildtype	61^b^	unaffected		
012	M	Wildtype	Wildtype	60^b^	unaffected		
013	F	unknown	unknown	NA	Uterine	NA	NA
014	M	Carrier	Carrier	56	CRC	Transverse colon	Adenocarcinoma
				71	Prostate	Prostate	NA
016	M	Wildtype	Wildtype	46	Lymphoma	Right neck lymph node	Follicular lymphoma
017	F	Wildtype	Wildtype	50^b^	unaffected		
018	M	Carrier	Wildtype	57	Laryngeal	Larynx	Squamous cell carcinoma
				58	Prostate	Prostate (Right lobe)	Adenocarcinoma
021	F	Wildtype	Wildtype	34	Cervical	Uterus cervix	NA
023	M	Wildtype	Wildtype	54^b^	unaffected		
024	F	Wildtype	Wildtype	84^b^	unaffected		
025	F	Obligate carrier	Obligate carrier	34	Breast	NA	NA
026	M	Carrier	Carrier	58^b^	unaffected		
027	M	Carrier	Carrier	57	Metastatic cancer of liver with unknown primary	Liver	NA
028	F	Wildtype	Carrier	54^b^	unaffected		
030	M	Wildtype	Wildtype	82^b^	unaffected		
034	F	unknown	unknown	NA	Intestinal	NA	NA
036	F	Wildtype	Wildtype	50	Endometrial	Uterus	Adenocarcinoma
042	F	Wildtype	Wildtype	44^b^	unaffected		
045	M	unknown	unknown	NA	Lung	NA	NA
047	F	unknown	unknown	NA	Kidney	NA	NA
055	M	Wildtype	NA ^c^	35^b^	unaffected		
056	F	Carrier	NA ^c^	NA	Breast	NA	NA
100	F	Carrier	NA	28^b^	unaffected		
101	F	Wildtype	NA	26^b^	unaffected		

^a^ cumulative serrated polyp history fulfils criteria for Serrated Polyposis Syndrome

^b^ age at last contact

^c^ clinical testing for the *BRCA1* variant only was undertaken

Abbreviations: NA, not available; CRC, colorectal cancer; F, female; M, male; SSL, sessile serrated lesion

The proband (person 001), a carrier of both the *BRCA1* and *RNF43* variants, was diagnosed with an adenocarcinoma of the caecum at age 53, a peritoneal cancer at age 62 and an ovarian cancer at age 63. MMR immunohistochemistry (IHC) of the metastatic lymph nodes indicated the CRC tumor was MMR-proficient. Three colonoscopies performed between the ages of 52 and 62 identified “numerous small metaplastic polyps” although the number and specific pathology were not reported, and, therefore, unclear if this person met the criteria for SPS. She died at age 67.

Two of the proband’s daughters (009 and 010) carried both the *BRCA1* and *RNF43* variants and both were CRC-affected. Person 009 was diagnosed with an MMR-proficient adenocarcinoma of the transverse colon at age 44. There was no report of synchronous polyps. Person 010 was diagnosed with a 15 mm moderately differentiated adenocarcinoma of the sigmoid colon at age 56, which appeared to have arisen from an SSL. Nine colonoscopy procedures between the ages of 40 and 63 revealed multiple serrated and adenomatous polyps. At the age of 56, a colonoscopy revealed a 10 mm hyperplastic polyp in the transverse colon in addition to the CRC. At the age of 59, a repeat colonoscopy showed a 6–8 mm adenomatous polyp and a 6–8 mm SSL in the ascending colon and two 6–8 mm hyperplastic polyps in the left colon. At the age of 62, a further colonoscopy showed a 5–8 mm hyperplastic polyp in the rectum. Including the SSL from which the adenocarcinoma had arisen from, person 010 met the 2019 WHO diagnostic criterion 1 for SPS [10].

Person 014 (a brother of the proband) was a carrier of the *BRCA1* and *RNF43* variants. He was diagnosed with an MMR-proficient adenocarcinoma of the transverse colon at age 56 and a prostate cancer at age 71. Person 018 (another brother of the proband) was a carrier of the *BRCA1* variant but not the *RNF43* variant. He was diagnosed with pharyngeal squamous cell carcinoma at age 57 and prostate adenocarcinoma at age 58. Two of the *BRCA1* carriers developed breast cancer (persons 025 and 056), one of whom was diagnosed at 34 years of age; the subtype was unavailable.

### Tumor analysis

**Fig. 2:**
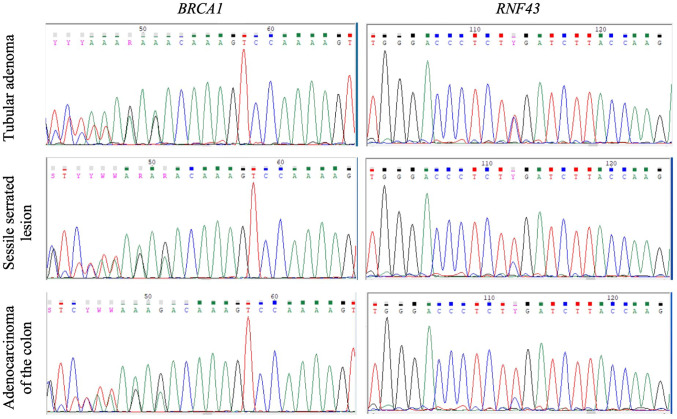
Sanger sequencing of the *BRCA1*:c.2681_2682delAA (left column) and *RNF43*:c.988 C>T (right column) pathogenic variants in a tubular adenoma, sessile serrated lesion (SSL), and colorectal cancer (CRC) for person 010 showing loss of heterozygosity (LOH) of the wildtype allele for both variants in the sessile serrated lesion and CRC but not the tubular adenoma

The CRCs from persons 009 and 014 were both MMR-proficient by IHC, wildtype for *BRAF* p.V600 and *KRAS* codon 12 and 13 somatic mutations and were CIMP-negative (Table 2) suggesting they had not developed via the serrated pathway of tumorigenesis. The MMR-proficient CRC and contiguous SSL from person 010 were both *BRAF* p.V600E mutation positive and CIMP-high, consistent with development via the serrated pathway (Table 2). Sanger sequencing of the *BRCA1* and *RNF43* variants in the tubular adenoma, SSL and CRC from person 010 showed evidence of LOH of the wildtype allele for both variants in the SSL and adenocarcinoma but not the tubular adenoma (Table 2; Fig. 2).

**Table 2:** Molecular characteristics of tumors from the family co-segregating the *BRCA1*:c.2681_2682delAA and *RNF43*:c.988 C>T germline pathogenic variants

Person	Germline BRCA1:c.2681_2682delAA	Germline RNF43:c.988 C > T	Sample description	Anatomical site	MMR status by IHC	LOH in BRCA1	LOH in RNF43	SBS mutational signature proportions	ID mutational signature proportions	CIMP status	BRAF p.V600E	KRAS codon 12 & 13	Somatic mutations
001	Carrier	Carrier	Lymph node metastasis from cecal cancer	Lymph node	Proficient	NA	NA	NA	NA	NA	NA	NA	
009	Carrier	Carrier	Adenocarcinoma	Transverse colon	Proficient	Yes^a^	Yes^a^	SBS3 (61.8%), SBS1 (11.3%), SBS30 (8.9%)	ID6 (65.2%), ID5 (30.5%), ID1 (4.3%)	Negative	Absent	WT	TP53 NM_000546.5:c.949del
010	Carrier	Carrier	Tubular adenoma	Ascending colon	Proficient	No	No	NA	NA	Negative	Absent	WT	
010	Carrier	Carrier	Sessile serrated lesion	Sigmoid colon	NA	Yes	Yes	NA	NA	Positive (high)	Present	WT	
010	Carrier	Carrier	Adenocarcinoma	Sigmoid colon	NA	Yes	Yes	NA	NA	Positive (high)	Present	WT	
014	Carrier	Carrier	Adenocarcinoma	Transverse colon	Proficient	NA	NA	NA	NA	Negative	Absent	WT	
018	Carrier	WT	Squamous cell carcinoma	Larynx	Proficient	NA	NA	NA	NA	NA	NA	NA	

^a^ Loss of heterozygosity (LOH) was determined from the whole exome sequencing data for this tumor

Abbreviations: WT, wild type; NA, no information/not tested; MMR, mismatch repair; IHC, immunohistochemistry; LOH, loss of heterozygosity; CIMP, CpG Island Methylator Phenotype, SBS, single base substitution; ID, insertion/deletion

**Fig. 3:**
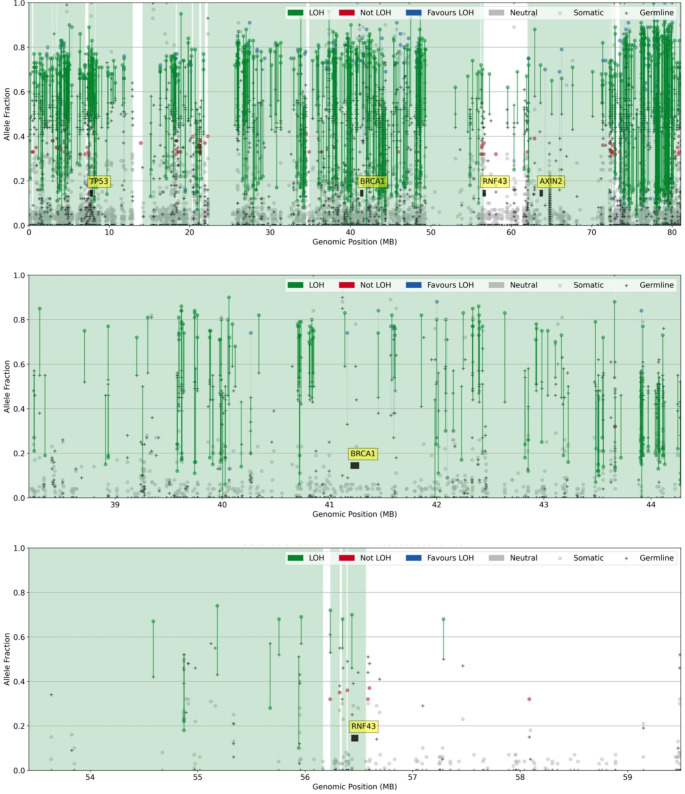
Allele frequency plot for a colorectal tumor of a person (person 009) with *BRCA1*:c.2681_2682delAA and *RNF43*:c.988 C>T germline pathogenic variants showing loss of heterozygosity across chromosome 17, including *BRCA1* and *RNF43*

**Fig. 4:**
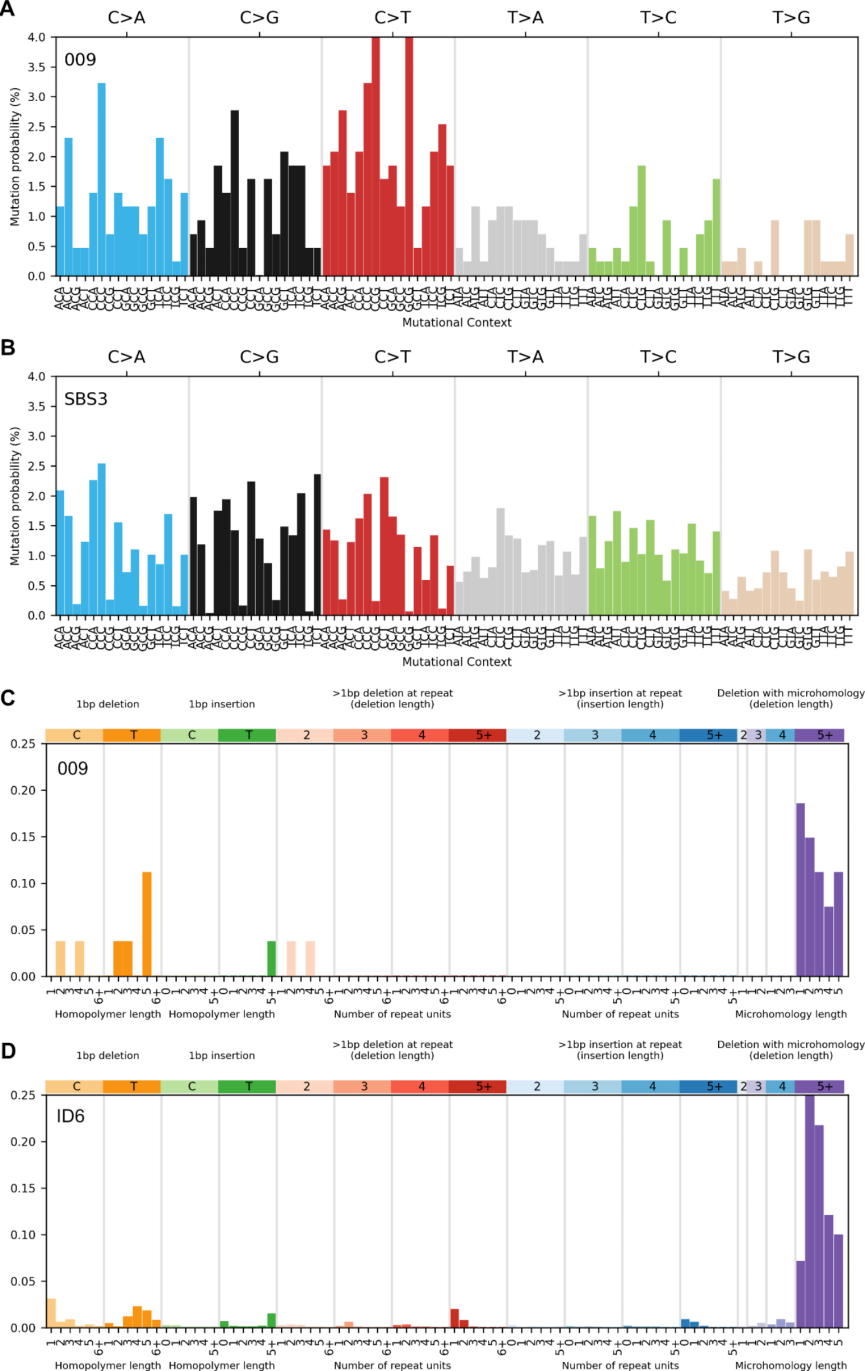
Comparing SNV-derived mutational contexts of a person with *BRCA1*:c.2681_2682delAA and *RNF43*:c.988 C > T germline pathogenic variants (person 009) **(A)** with defective homologous recombination-based DNA damage repair associated signature SBS3 **(B)**, and similarly, indel-derived contexts of person 009 **(C)** with ID6 **(D)**

To further investigate tumor etiology, the CRC from person 009 underwent WES. No somatic mutations in *BRCA1* and *RNF43* were observed, however, loss of the wildtype allele was evident for both variants. LOH of a larger region across chromosome arm 17q was detected that included the *BRCA1* and *RNF43* genes (Fig. 3). Analysis of COSMIC tumor mutational signature profiles revealed SBS3 (61.8%), SBS1 (11.3%) and SBS30 (8.9%) as the SNV-derived signatures with the highest proportion. The observed indels in this tumor were decomposed into the signatures ID6 (65.2%), ID5 (30.5%) and ID1 (4.3%), with the predominance of both SBS3 and ID6 indicative of defective homologous recombination-based DNA damage repair (HRD); the contexts of SBS3 and ID6 are compared to those observed in 009 in Fig. 4.

The top plot covers the whole of chromosome 17. The middle plot covers a region around *BRCA1*. The bottom plot covers a region around *RNF43*.

## Discussion

This study identifies a family meeting the criteria for FCCTX where a germline *BRCA1*:c.2681_2682delAA p.Lys894ThrfsTer8 pathogenic variant and a germline *RNF43*:c.988 C>T p.Arg330Ter pathogenic variant co-segregated with CRC in four carriers, one of whom was confirmed to meet the WHO^2019^ diagnostic criteria 1 for SPS. Tumor analysis demonstrated loss of the wildtype allele for both variants in the two CRCs tested. As both *BRCA1* and *RNF43* reside on chromosome 17q, the LOH observed across the region encompassing both these tumor suppressor genes (Fig. 3) confirms that both *BRCA1* and *RNF43* had biallelic inactivation. The presence of both the tumor mutational signatures SBS3 and ID6 at high levels (> 50%), which is associated with HRD, and the absence of serrated pathway molecular characteristics, namely the *BRAF* p.V600E mutation and CIMP-high, suggests that tumorigenesis for the CRC from person 009 was driven by HRD deficiency related to *BRCA1* inactivation. In contrast, biallelic inactivation of *BRCA1* and *RNF43* was also present in the CRC from person 010 with the tumor demonstrating characteristics of the serrated pathway (*BRAF* p.V600E mutation and high levels of CIMP), suggesting that for this tumour tumorigenesis may have been driven by *RNF43* deficiency.

Germline pathogenic variants in *BRCA1* predispose carriers to significantly elevated risks of breast and ovarian cancers [46], but the relationship between *BRCA1* and CRC susceptibility is less clear [22]. A recent meta-analysis and systematic review showed *BRCA1* and/or *BRCA2* pathogenic variant carriers did not have a higher risk of developing CRC [47]. Past studies have suggested *BRCA2* may underlie CRC development in FCCTX families, however, there is little evidence implicating *BRCA1* [48, 49]. In the current study, ten family members carried the *BRCA1* variant, four developing CRC with only three developing a breast or ovarian cancer. Tumor WES derived analysis from the single CRC from person 009 demonstrated that tumorigenesis was dominated by the *BRCA1* variant-related HRD process, evidenced by the high proportion of HRD-related SBS3 and ID6 mutational signatures. Despite this, it is possible that *RNF43* deficiency has also contributed to the initiation and/or progression of tumorigenesis in this person together with HRD.

Somatic mutations in *RNF43* play a role in colorectal tumorigenesis including in the serrated pathway [50–54]. Furthermore, although rare in SPS [19], several studies have now provided evidence that germline *RNF43* variants are associated with SPS [14–19, 53]. Only a few of these studies have investigated segregation of the *RNF43* variant with SPS in the family. Of note, a study by Taupin et al. [15] identified a germline nonsense variant in *RNF43* (c.394 C>T p.Arg132Ter) in two siblings affected with SPS, one developed CRC and a study by Yan et al. [17] identified a germline splice site variant (c.953-1 G>A) in *RNF43* carried by six people from the family. Five of the six carriers met the WHO^2010^ criteria for SPS with a second somatic hit in *RNF43* (predominantly LOH) identified in all 22 cancers/polyps analyzed [17]. There were eight carriers of the *RNF43*:c.988 C>T p.Arg330Ter variant in the family from this study, four were CRC-affected and a single carrier was confirmed to meet the WHO^2019^ criteria for SPS. Furthermore, LOH was observed as the second somatic hit in both CRCs tested and in an SSL polyp. [16, 17] Our findings add further support for the association between germline *RNF43* variants and susceptibility to SPS and CRC.

Tumor mutational signature analysis is an important tool for understanding tumor etiology and for predicting response to cancer therapies, including the use of PARP inhibitors for cancers with HRD [55]. Of the current COSMIC mutational signatures, SBS3 and ID6 are associated with HRD, which are associated with defects in *BRCA1*, *BRCA2* or other genes involved in the homologous recombination pathway [55, 56], although HRD in CRC is not commonly observed [57]. In the CRC from person 009, both SBS3 and ID6 were the dominant mutational signatures, supporting HRD related to the germline *BRCA1* variant.

This study has several limitations. Phenotype data was not available from all family members including incomplete or historic colonoscopy and/or pathology reports that meant some of the colonic polyp number and morphological classification was not definitive or equivalent to contemporary polyp classification. Little data was obtained from earlier generations as those generations were deceased prior to commencing a detailed investigation. Furthermore, the tumor tissue for molecular testing was limited with only a single CRC with sufficient DNA for WES and therefore, confirmation that HRD associated mutational signatures were the dominant mutational process in the other CRCs from *BRCA1* carriers could not be determined. Further investigation of HRD in CRC tumorigenesis is needed.

## Conclusion

In summary, we have identified coinheritance of pathogenic germline variants in *BRCA1* and *RNF43* segregating with CRC in a family previously characterized as FCCTX. One individual satisfied the diagnostic criteria for SPS, and there was evidence for a somatic second-hit in *BRCA1* and *RNF43* in the form of LOH. Bioinformatic analysis showed that the tumorigenesis was predominantly driven by the *BRCA1* variant with LOH, as indicated by the HRD-related mutational signatures in the tumor. Our study highlights a possible role of digenic inheritance underlying FCCTX.
